# The Loss of PGAM5 Suppresses the Mitochondrial Degeneration Caused by Inactivation of PINK1 in *Drosophila*

**DOI:** 10.1371/journal.pgen.1001229

**Published:** 2010-12-02

**Authors:** Yuzuru Imai, Tomoko Kanao, Tomoyo Sawada, Yoshito Kobayashi, Yasuhiro Moriwaki, Yosuke Ishida, Kohsuke Takeda, Hidenori Ichijo, Bingwei Lu, Ryosuke Takahashi

**Affiliations:** 1 Institute of Development, Aging, and Cancer, Tohoku University, Sendai, Japan; 2 Department of Neurology, Kyoto University Graduate School of Medicine, Kyoto, Japan; 3 Department of Pharmacology, Faculty of Pharmacy, Keio University, Tokyo, Japan; 4 Laboratory of Cell Signaling, Graduate School of Pharmaceutical Sciences, The University of Tokyo, Tokyo, Japan; 5 Department of Pathology, Stanford University School of Medicine, Stanford, California, United States of America; Baylor College of Medicine, United States of America

## Abstract

PTEN-induced kinase 1 (PINK1), which is required for mitochondrial homeostasis, is a gene product responsible for early-onset Parkinson's disease (PD). Another early onset PD gene product, Parkin, has been suggested to function downstream of the PINK1 signalling pathway based on genetic studies in *Drosophila*. PINK1 is a serine/threonine kinase with a predicted mitochondrial target sequence and a probable transmembrane domain at the N-terminus, while Parkin is a RING-finger protein with ubiquitin-ligase (E3) activity. However, how PINK1 and Parkin regulate mitochondrial activity is largely unknown. To explore the molecular mechanism underlying the interaction between PINK1 and Parkin, we biochemically purified PINK1-binding proteins from human cultured cells and screened the genes encoding these binding proteins using *Drosophila* PINK1 (dPINK1) models to isolate a molecule(s) involved in the PINK1 pathology. Here we report that a PINK1-binding mitochondrial protein, PGAM5, modulates the PINK1 pathway. Loss of *Drosophila* PGAM5 (dPGAM5) can suppress the muscle degeneration, motor defects, and shorter lifespan that result from dPINK1 inactivation and that can be attributed to mitochondrial degeneration. However, dPGAM5 inactivation fails to modulate the phenotypes of *parkin* mutant flies. Conversely, ectopic expression of dPGAM5 exacerbated the *dPINK1* and *Drosophila parkin* (*dParkin*) phenotypes. These results suggest that PGAM5 negatively regulates the PINK1 pathway related to maintenance of the mitochondria and, furthermore, that PGAM5 acts between PINK1 and Parkin, or functions independently of Parkin downstream of PINK1.

## Author Summary

Parkinson's disease (PD) is a neurodegenerative disease pathologically characterized by degeneration of dopaminergic (DA) neurons in the midbrain. A small percentage of PD cases are inherited in a Mendelian manner, and several disease-causing genes have been identified. The *PINK1* and *Parkin* genes have been isolated as the genes for autosomal recessive form of early-onset PD. Unexpectedly, loss of function of either PINK1 or Parkin in *Drosophila* causes mitochondrial degeneration in the flight muscles, which exhibits a visible phenotype of abnormal wing postures, allowing a rapid genetic screening. We purified PINK1-binding proteins from human cultured cells and screened the gene for these binding proteins using the *PINK1* mutant flies. We found that inactivation of a PINK1-binding protein phosphoglycerate mutase 5 (PGAM5) suppresses mitochondrial degeneration caused by the loss of PINK1 activity. Although *parkin* is suggested to be genetically downstream of *PINK1* in *Drosophila*, loss of PGAM5 failed to modulate the phenotypes by *parkin* inactivation. Our finding suggested that, for mitochondrial maintenance of tissues with high-energy demands such as the muscles and DA neurons, PGAM5 acts between PINK1 and Parkin, or functions independently of Parkin downstream of PINK1.

## Introduction

Parkinson's disease (PD (OMIM #168600)) is a neurodegenerative disease that affects the maintenance of dopaminergic (DA) neurons. PD prevalence is estimated at ∼1% among people over the age of 65 and increases with age. Clinical features of PD include motor abnormalities (tremor, rigidity, akinesia), autonomic disturbances, psychiatric disability and cognitive impairment. The recent identification of PD-associated genes has advanced our understanding the molecular mechanisms underlying PD. Two of these genes, *PINK1* (PARK6, OMIM #605909, Gene ID: 65018) and *parkin* (PARK2, OMIM #600116, Gene ID: 5071), are associated with early-onset autosomal recessive PD, in which loss-of-function (LOF) of a single gene product results in the clinical manifestation of Parkinsonism [1], [2]. The *PINK1* gene encodes a serine/threonine kinase with a predicted mitochondrial target sequence and a probable transmembrane domain at the N-terminus [3]. The gene product of the *parkin* gene encodes a protein with an E3 activity [4]–[6]. Recent genetic studies in *Drosophila* have reported that *dPINK1* (Gene ID: 31607) acts as an upstream regulator of *dParkin* (Gene ID: 40336) in a common pathway that influences mitochondrial maintenance in a subset of tissues, including the flight muscle and DA neurons [7]–[9]. LOF of the *dPINK1* or the *dparkin* genes results in enlarged or swollen mitochondria, a phenotype that can be partially rescued by heterozygosity for LOF mutations of the mitochondrial fusion-promoting components Optic atrophy 1 (OPA1) and Mitofusin (Mfn), or by increased mitochondrial fission activity via increased dosage of the *dynamin-related protein 1* (*drp1*) gene [10]–[12]. Studies in mammalian or *Drosophila* cultured cells report that PINK1 is required to recruit Parkin to damaged depolarized mitochondria, and promotes their degradation through an autophagic event called mitophagy [13]–[16]. Thus, there is strong evidence to support an important role for PINK1 and Parkin in regulating mitochondrial homeostasis. However, little is known about how PINK1 regulates mitochondrial integrity and turnover through Parkin. Indeed, the precise means by which PINK1 exerts an effect on Parkin is not clear.

Here we show that a mitochondrial protein, phosphoglycerate mutase 5 (PGAM5, Gene ID: 192111), which was previously reported to be localized at the outer mitochondrial membrane and to lack a phosphoglycerate mutase activity [17], [18], is involved in the PINK1 pathway, and that loss of PGAM5 activity improves mitochondrial defects caused by PINK1 inactivation in *Drosophila*.

## Results

### Isolation of PGAM5 as a PINK1-Binding Protein

**Figure 1:**
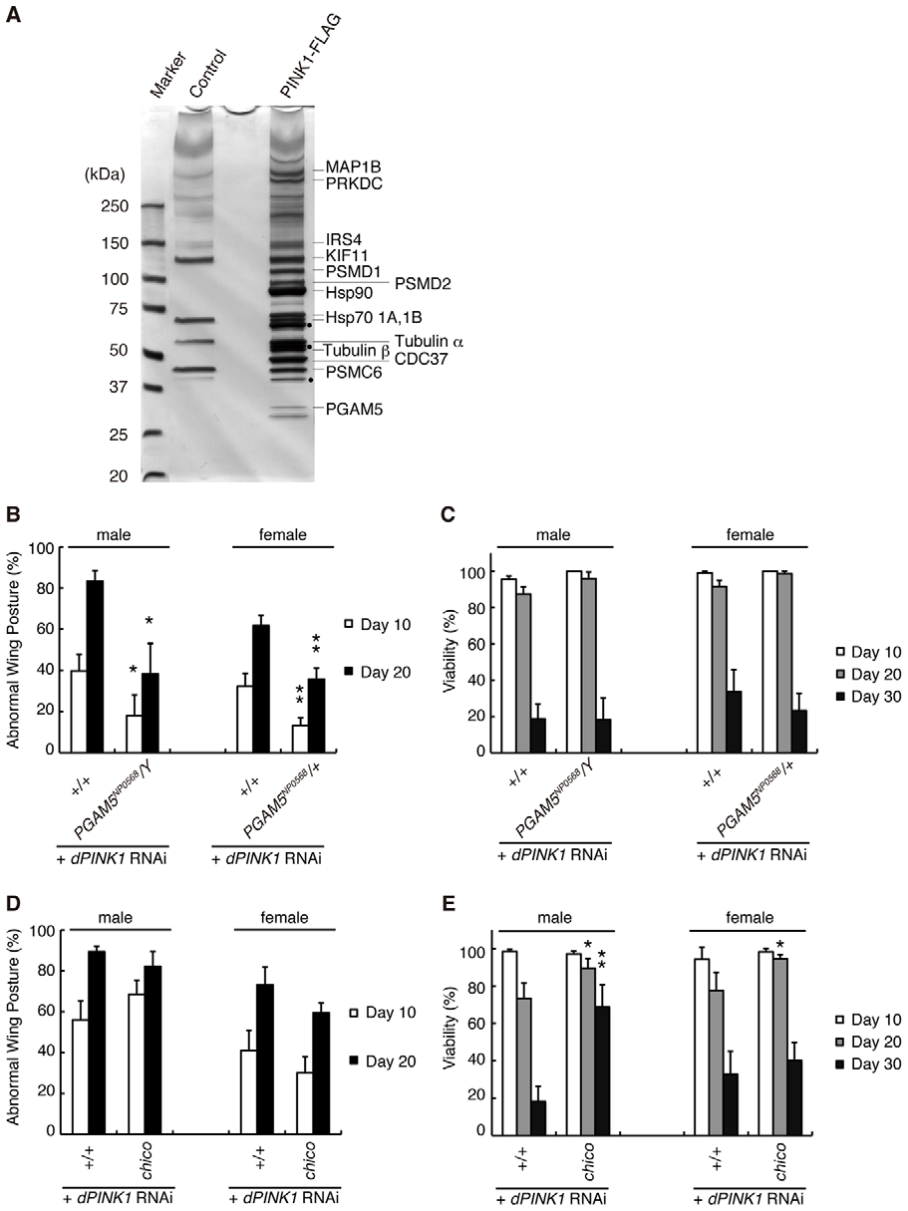
Identification of PINK1-binding proteins that modulate the phenotypes of *dPINK1* knockdown fly. (A) Silver-stained polyacrylamide gel to visualize hPINK1-binding proteins. FLAG elution fractions purified from cells stably expressing hPINK1-FLAG (PINK1-FLAG lane) and parental cells (Control lane) are separated on a gel (For details of the procedure, see Materials and Methods). Bands corresponding to hPINK1 (dots) and representative co-purified proteins are indicated. (B, C) The wing phenotype typical of 10- and 20-day-old *dPINK1* RNAi flies [9] (B) was suppressed by the *PGAM5^NP0568^* mutant allele, whereas viability of 10-, 20- and 30-day-old adult flies was not improved (C). *, *p*<0.05; **, *p*<0.01 *vs.* age-matched *dPINK1* RNAi group in Student's *t*-test. The genotypes are as follows: *MHC-GAL4> dPINK1^RNAi^* (+/+), *PGAM5^NP0568^/Y; MHC-GAL4> dPINK1^RNAi^* (*PGAM5^NP0568^/Y*), *PGAM5^NP0568^/+; MHC-GAL4> dPINK1^RNAi^* (*PGAM5^NP0568^/+*). *MHC-GAL4*, a muscle-specific driver. Flies were raised at 29°C as the RNAi-induced dPINK1 defects are more pronounced when flies are raised at that temperature. (D, E) Removal of one copy of the *IRS4* ortholog *chico* had no effect on the wing phenotype of *dPINK1* RNAi flies (D) but improved viability (E). *, *p*<0.05; **, *p*<0.01 *vs.* age-matched *dPINK1* RNAi group. The genotypes are: *MHC-GAL4> dPINK1^RNAi^* (+/+), *chico*/+; *MHC-GAL4> dPINK1^RNAi^* (*chico*). Flies were raised at 29°C.

We and others have previously demonstrated that *PINK1* is genetically upstream of *parkin* [7]–[9]. To further investigate the relationship between PINK1 and Parkin, we searched for PINK1-binding proteins using a combination of biochemical purification and mass spectrometric analysis. We affinity-purified human PINK1 with a FLAG tag at its C-terminus (hPINK1-FLAG) from lysate of human embryonic kidney (HEK) 293 cells stably expressing hPINK1-FLAG using an anti-FLAG column, and determined proteins specifically presented in the hPINK1-FLAG elution fractions, which include cytoskeleton-related proteins (MAP1B (GeneID: 4131), KIF11 (GeneID: 3832), Tubulin GeneID: 602530, 191130)), proteasome subunits (PSMD1 (GeneID: 5707), PSMD2 (GeneID: 5708), PSMC6 (GeneID: 5706)), PRKDC (GeneID: 5591), Hsp70 (1A, GeneID: 3303; 1B, GeneID: 3304), Hsp90 (GeneID: 3320), Cdc37 (GeneID: 11140), Insulin substrate-4 (IRS-4, GeneID: 8471) and PGAM5 (Figure 1A). PRKDC is one of proteins non-specifically associated with FLAG-tagged proteins in our proteomic analyses (data not shown). The roles of Hsp90, Cdc37 and the proteasome for PINK1 have been characterized previously [15], [19]–[22]. We therefore chose IRS-4 and PGAM5 and tested whether these proteins modulate the *dPINK1* LOF phenotypes by *Drosophila* genetics. Our initial *in vivo* tests revealed that a mutant allele for *dPGAM5* (*CG14816*, GeneID: 31143), *PGAM5^NP0568^* significantly suppressed the abnormal wing postures observed in *dPINK1* knockdown flies [9] (Figure 1B), while it failed to improve the viability (Figure 1C). Reducing the dose of *chico* (GeneID: 64880), which encodes a *Drosophila* orthologue of IRS-4, significantly suppresses the short lifespan phenotype caused by *dPINK1* knockdown, without affecting wing posture (Figure 1D and 1E). Inhibition of *chico* activity has previously been reported to extend the lifespan of *Drosophila*, such that we reasoned that the effect on lifespan we observed might reflect a general phenomenon rather than reflecting a specific interaction with *dPINK1* [23]. Thus for subsequent studies, we chose to focus on PGAM5.

**Figure 2:**
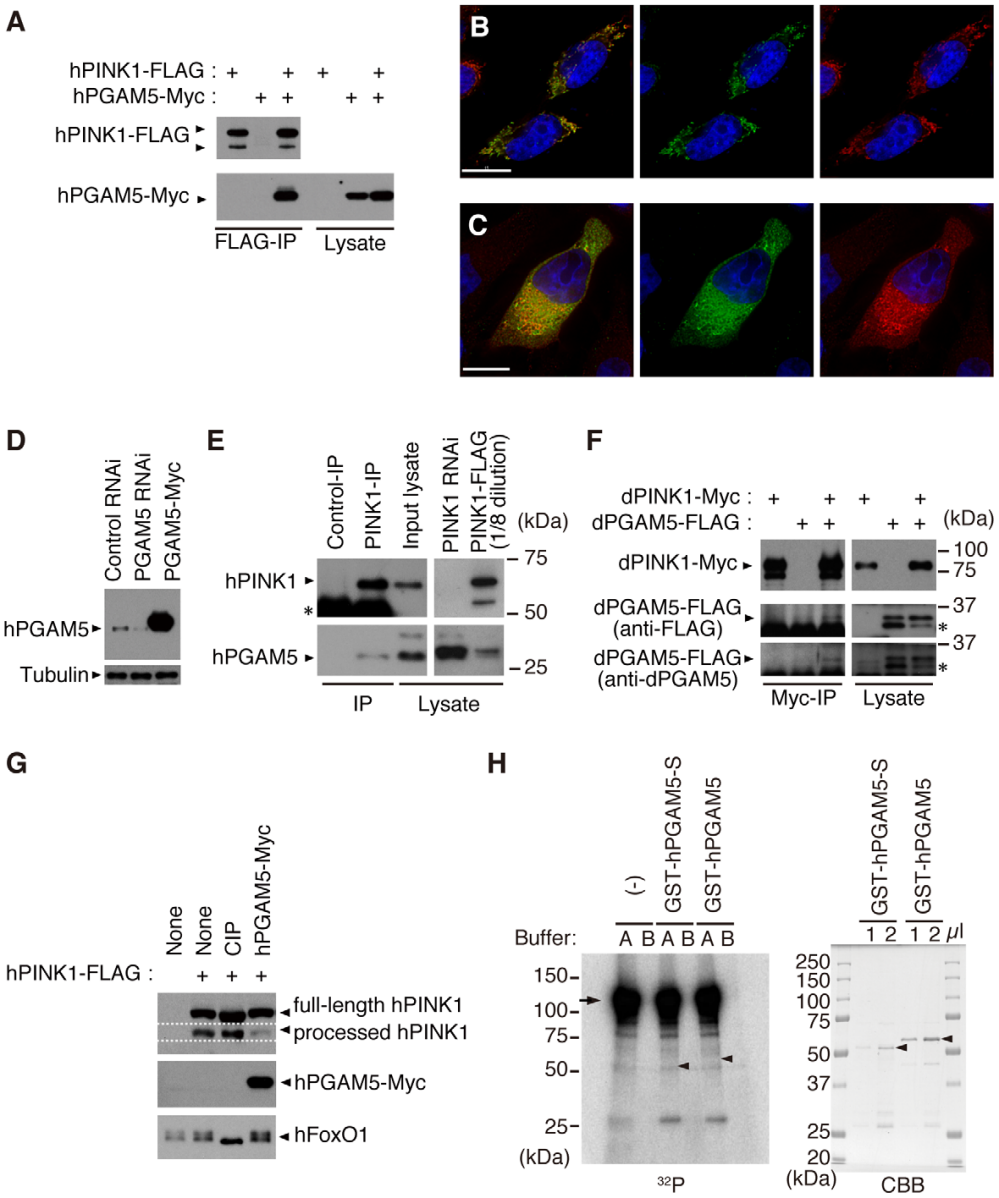
PGAM5 associates with PINK1 at mitochondria. (A) hPGAM5 binds to hPINK1 in HEK293 cells. Lysate expressing C-terminally Myc-tagged hPGAM5 (hPGAM5-Myc) and FLAG-tagged hPINK1 (hPINK1-FLAG) was subjected to immunoprecipitation with anti-FLAG antibody (FLAG-IP), and analyzed by immunoblotting with anti-tag antibodies. (B) hPGAM5 is localized to the mitochondria. HeLa cells transfected with hPGAM5-Myc were visualized with anti-Myc (green). Mitochondria were visualized with MitoTracker (red) and nuclei with DAPI (blue). Regions of co-localization of hPGAM5 with mitochondria appear in yellow in the merged image. (C) hPGAM5 and hPINK1 co-localize at mitochondria. HeLa cells co-transfected with hPINK1 and hPGAM5-Myc were stained with anti-PINK1 (green) and anti-Myc (red). (D) Anti-hPGAM5 antibody specifically recognizes ∼30 kDa bands in extract from HEK293 cells, which were reduced in lysates from cells treated with siRNAs directed against hPGAM5. Lysate expressing hPGAM5-Myc and anti-tubulin signals served as a positive control and a loading control, respectively. (E) Endogenous hPGAM5 is associated with hPINK1. An anti-PINK1 (PINK1-IP) or an antibody against the unrelated protein Delta (Control-IP) was used for immunoprecipitation of proteins in HEK293 cells. Cell lysate in which hPINK1 was knocked down by RNAi (PINK1 RNAi) and lysate from cells that overexpressed hPINK1-FLAG (PINK1-FLAG) served as additional controls. The PINK1-FLAG lysate was diluted eight-fold with loading buffer to reduce the strong signal present in that sample. Asterisk, bands attributable to detection of the antibodies themselves, which may mask lower molecular weight hPINK1 bands (∼52 kDa). (F) dPGAM5 is associated with dPINK1 in *Drosophila* S2 cells. S2 cell lysate expressing dPINK1-Myc and dPGAM5-FLAG was subjected to immunoprecipitation with anti-Myc antibody (Myc-IP), and analyzed by immunoblotting with anti-tag or anti-dPGAM5 antibodies. Asterisks, a putative processed form of dPGAM5. (G) HEK 293 cell lysate expressing hPINK1-FLAG together with hPGAM5-Myc was subjected to Phos-tag immunoblotting [43]. hPINK1-FLAG lysate treated with alkaline phosphatase (CIP) was used as a positive control. A phospho-protein FoxO1 was efficiently dephosphorylated by the CIP treatment. (H) An *in vitro* kinase assay was performed using 2x GST-dPINK1 and GST-hPGAM5. Recombinant 2x GST-dPINK1 purified from bacteria was used as a kinase source. Recombinant GST-hPGAM5 short form (GST-hPGAM5-S) or GST-hPGAM5 was purified from bacteria and 1 and 2 µl of the purified fractions were separated by SDS-PAGE and stained with Coomassie Brilliant Blue (CBB, right-hand panel; arrowheads, GST-hPGAM5-S or GST-hPGAM5). A total of 100 or 400 ng of GST-hPGAM5-S or GST-hPGAM5, respectively, were incubated with 100 ng of 2x GST-dPINK1 in kinase reaction buffer A (100 mM Tris-HCl [pH 7.5], 240 mM NaCl, 30 µM ATP, 10 mM MgCl_2_, 2 mM CaCl_2_, 5 µCi γ-^32^P ATP) or buffer B (100 mM Tris-HCl [pH7.5], 240 mM NaCl, 30 µM ATP, 10 mM EDTA, 5 µCi γ-^32^P ATP) for 30 min at 30°C. The reaction mixture was suspended in SDS sample buffer and then subjected to SDS-PAGE and autoradiography (Left, ^32^P; the arrow and arrowheads represent expected migration positions of 2x GST-dPINK1 and GST-hPGAM5/GST-hPGAM5-S, respectively). No specific signals corresponding hPGAM5 or hPGAM5-S were observed. Note that 2x GST-dPINK1 lacks kinase activity in the buffer B, suggesting that activation of PINK1 requires divalent cations such as Mg^2+^ and Ca^2+^. Scale bars  = 15 µm in (B and C).

The results of co-immunoprecipitation confirmed that C-terminally Myc-tagged human PGAM5 (hPGAM5-Myc) specifically binds to hPINK1-FLAG in transfected HEK293 cells (Figure 2A). Moreover, we found that hPGAM5 and hPINK1 immunoreactivity co-localizes with mitochondria in transfected HeLa cells, consistent with the previous finding that PGAM5 is localized to the mitochondria (Figure 2B and 2C) [24]. To test if endogenous hPGAM5 interacts with hPINK1, we first generated an anti-hPGAM5 antibody (Figure 2D). Next, we used a previously established anti-PINK1 antibody to immunoprecipitate PINK1 from HEK293 cell lysate, then probed with anti-PGAM5 to detect endogenous hPGAM5. As shown in Figure 2E, endogenous hPGAM5 was detectable in the fraction immunoprecipitated using anti-hPINK1 antibody but not a control antibody, confirming the results of mass spectrometric analysis. Physical association of dPINK1 with dPGAM5 was also observed in *Drosophila* S2 cells (Figure 2F), suggesting that their functional interaction is conserved between human and *Drosophila*.

Previous findings that PINK1 and PGAM5 possess kinase and phosphatase activities, respectively [18], [25], [26], prompted us to test the possibility of their enzyme-substrate relationships. A mobility shift assay to monitor the status of PINK1 phosphorylation suggested that overexpression of hPGMA5 has little effect on hPINK1 phosphorylation (Figure 2G). On the other hand, an *in vitro* kinase assay using recombinant dPINK1 failed to show a possibility that PGAM5 is a substrate for PINK1, or that PGAM5 modifies hPINK1 kinase activities (Figure 2H).

### dPGAM5 Alters Mitochondrial Morphology in *Drosophila*

The *Drosophila* genome appears to have two orthologs of mammalian *PGAM5*, one on the X (*CG14816*) and the other on the second chromosome (*CG15874*, GeneID: 37899). We have renamed *CG14816* and *CG15874* as *dPGAM5* and *dPGAM5-2*, respectively. Our initial *in vivo* genetic study and most subsequent analyses were performed using *dPGAM5* mutant and transgenic animals because *dPGAM5* is more similar to *hPGAM5* than is *dPGAM5-2* (*dPGAM5 vs. hPGAM5*, 44% amino acid identity, and *dPGAM5-2 vs. hPGAM5*, 38% identity, as determined using ClustalW v1.4 to align the sequences), and because the results of high-throughput analysis of transcript abundance suggest that the *dPGAM5-2* transcript is expressed at very low levels at the adult stage, if at all (see http://flybase.org/reports/FBgn0035004.html).

**Figure 3:**
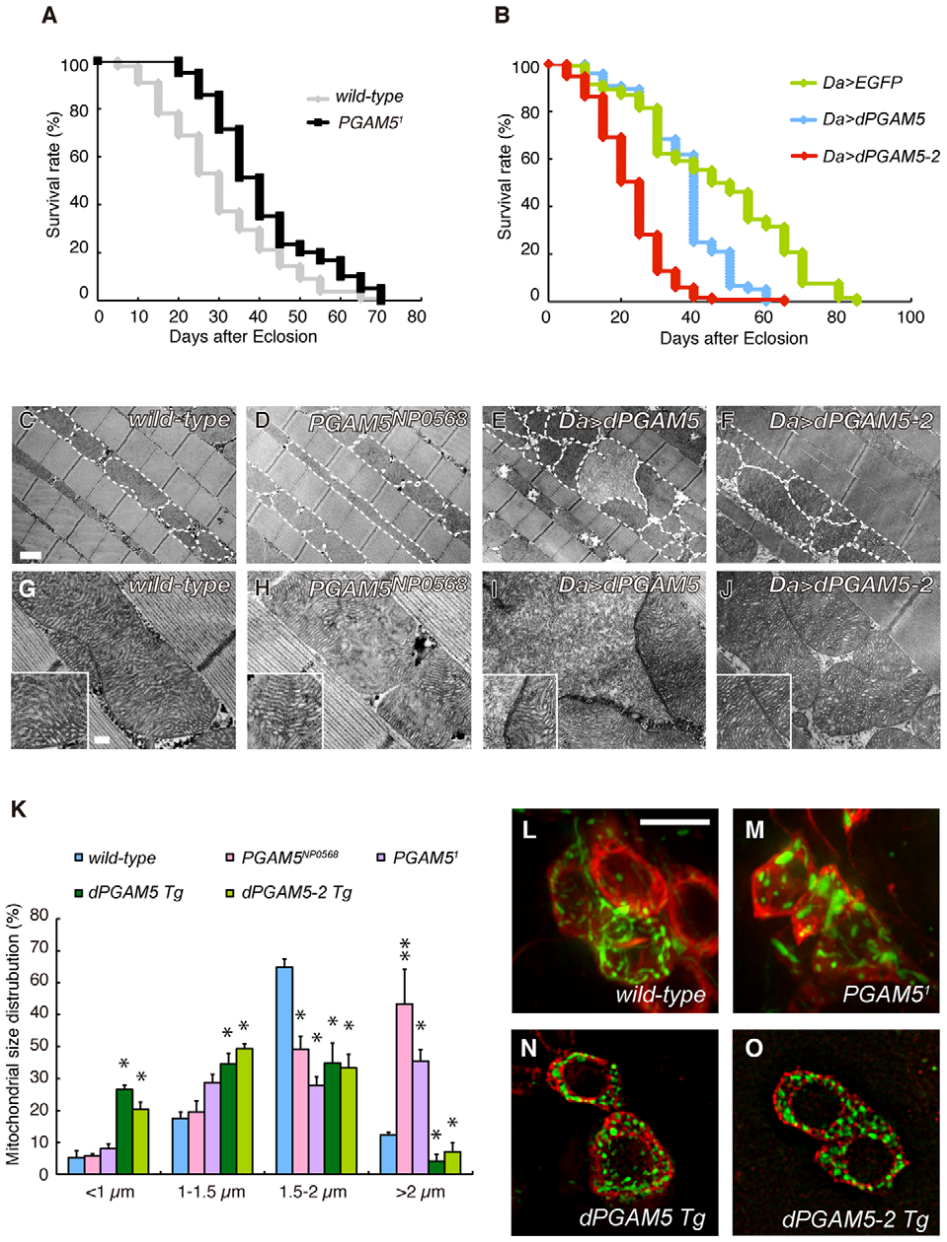
dPGAM5 is dispensable for normal development, but affects lifespan in *Drosophila*. (A) Loss of dPGAM5 genes extends the lifespan. Adult male *wild-type* (*yw/Y*; *n* = 125) *vs. dPGAM5 null* (*y*, *PGAM5^1^/Y; n* = 125) flies, *p*<0.001 by log rank test. (B) Overexpression of *dPGAM5* or *dPGAM5-2* in *Drosophila* causes shorter lifespan. Overexpression of the transgenes was induced using the ubiquitous *daughterless* (*Da*)*-GAL4* driver. Lifespan of adult male *EGFP* (*n* = 130), *dPGAM5* (*n* = 76) and *dPGAM5-2* (*n* = 117) flies. *EGFP vs. dPGAM5*, *p*<0.001; *EGFP vs. dPGAM5-2*, *p*<0.001; by log rank test. (C–J) Transmission electron microscopy (TEM) analysis of the indirect flight muscle and morphology of mitochondria in 2-day-old adult flies with the indicated genotypes. In C–F, we outlined some mitochondria with broken lines to highlight morphology. The insets in G–J show representative mitochondria matrixes. A revertant, *PINK1^RV^*, was used as a wild-type comparison [8]. The genotypes are: *PINK1^RV^/Y* (C, G), *PGAM5^NP0568^/Y* (D, H), *Da-GAL4> UAS*-*dPGAM5* (E, I), *Da-GAL4> UAS*-*dPGAM5-2* (F, J). Scale bars  = 1 µm in C–F and 200 nm in G–J. (K) Quantification of the percentage of mitochondrial size distribution in the indirect muscle tissue from *wild-type* (*n* = 136 from 5 adult flies), *PGAM5^NP0568^* (*n* = 155 from 5), *PGAM5^1^* (*n* = 87 from 5), *dPGAM5 Tg* (*n* = 143 from 5) and *dPGAM5-2 Tg* flies (*n* = 147 from 5) as shown in (C–J). The length of the mitochondria in the direction of the myofibrils was measured. Data are shown as means ± SE (* *p*<0.05, **p<0.01 *vs. wild-type*). (L–O) Brain tissues of 5-day-old adult flies were stained with anti-TH antibody (red). Mitochondria labeled with mitoGFP (green) were observed in the PPL1 TH-positive neurons of the indicated genotypes. The genotypes are as follows: *TH-GAL4> mitoGFP* (*wild-type*), *PGAM5^1^/Y; TH-GAL4>UAS-mitoGFP* (*PGAM5^1^*), *UAS-dPGAM5/TH-GAL4> UAS-mitoGFP* (*dPGAM5 Tg*), *UAS-dPGAM5-2/TH-GAL4> UAS-mitoGFP* (*dPGAM5-2 Tg*). *TH-GAL4*, a DA neuron-specific driver. Scale bar  = 5 µm.

We determined the *P*-element insertion allele *PGAM5^NP0568^* as a hypomorph allele, which showed a reduction of *dPGAM5* transcript levels to about 25% of normal levels (Figure S1). We then generated a *dPGAM5* null allele *PGAM5^1^*, in which the expression of dPGAM5 completely disappeared at both the transcript and protein levels (Figure S1B and S1C). The *PGAM5^1^* homozygous animal is viable, fertile and grossly normal. However, it displayed longer lifespan (Figure 3A). By contrast, overexpression of dPGAM5 or dPGAM5-2 resulted in shorter longevity (Figure 3B). Since a previous report described that overexpression of human PGAM5 affects the mitochondrial morphology or mobility in the cultured cells [24], we observed the mitochondria in the *dPGAM5* null and transgenic flies. Although inactivation of *dPGAM5* gene function did not cause mitochondrial degeneration, the morphology of the mitochondria appears to be moderately altered (Figure 3D and 3H compared to Figure 3C and 3G). The mitochondria in the indirect flight muscles of the *PGAM5* mutant flies were longer in the long-axis direction compared to control animals (Figure 3K). A similar tendency was seen in DA neurons of the adult brain although the difference did not reach statistical significance (Figure 3L and 3M). In addition, we frequently observed constrictions in the mitochondria (see broken lines in Figure 3D). In contrast, transgenic expression of dPGAM5 or dPGAM5-2 in *Drosophila* leads to fragmentation of mitochondria, with cristae well-preserved in the indirect flight muscles (Figure 3E, 3F, 3I, and 3J) and in the tyrosine-hydroxylase (TH)-positive neurons of the adult fly brain (Figure 3N and 3O). These results suggested that dPGAM5 is likely to promote the mitochondrial fission process in *Drosophila*.

### The Relationship between PGAM5 and the Mitochondrial Fission/Fusion Machinery

**Figure 4:**
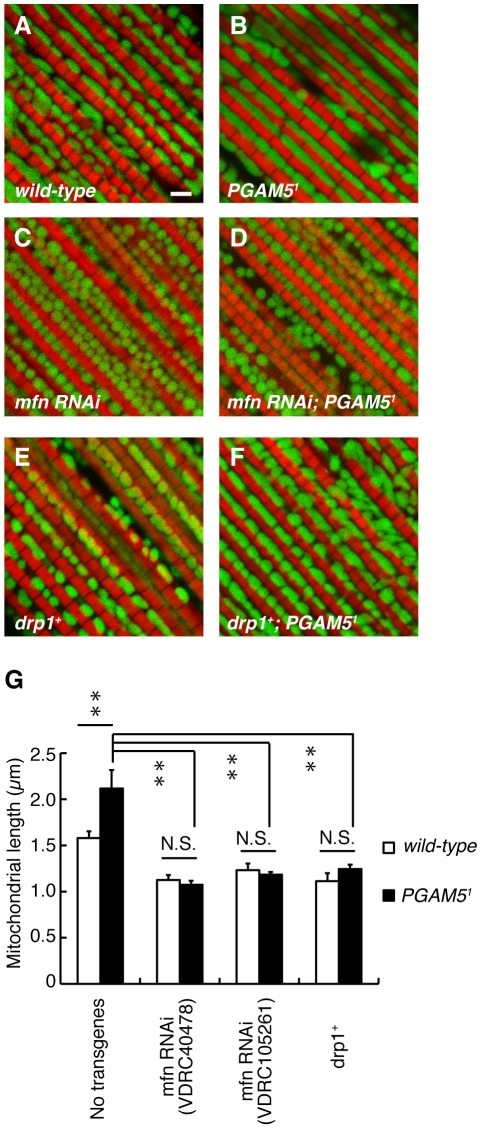
Relationship between *dPGAM5* and the mitochondrial fusion/fission genes. (A–F) dPGAM5 inactivation failed to rescue the mitochondrial fragmentation caused by *mfn* knockdown (*mfn RNAi*) or introduction of an extra copy of the *drp1* gene (*drp1^+^*). To visualize the mitochondria under a fluorescence microscopy, we used the muscle-specific *MHC-GAL4* driver to induce expression of a mitoGFP (green) transgene in 5-day-old adult flies with the indicated genotypes. Muscle tissue was counterstained with phalloidin (red). Scale bar  = 2 µm. (G) The average length of the mitochondria in the direction of the myofibrils was measured from *wild-type* (*n* = 343 from 7 adult flies), *PGAM5^1^* (*n* = 390 from 8), *mfn RNAi* (VDRC40478, *n* = 305 from 6; VDRC105261, *n* = 372 from 8), *mfn RNAi* (VDRC40478)*; PGAM5^1^* (*n* = 355 from 7), *mfn RNAi* (VDRC105261)*; PGAM5^1^* (*n* = 237 from 5), *drp1^+^* (*n* = 245 from 5) and *drp1^+^; PGAM5^1^* (*n* = 247 from 5) as shown in (A–F). Data are shown as means ± SE (**p<0.01; N.S., not significant). The genotypes are as follows: *+/Y; MHC-GAL4>mitoGFP* (A, *wild-type*), *PGAM5^1^/Y; MHC-GAL4>UAS-mitoGFP* (B, *PGAM5^1^*), *+/Y; MHC-GAL4>UAS-mitoGFP; UAS-mfn RNAi* (VDRC40478) (C, *mfn RNAi*), *PGAM5^1^/Y; MHC-GAL4>UAS-mitoGFP; UAS-mfn RNAi* (VDRC40478) (D, *mfn RNAi; PGAM5^1^*), *+/Y; MHC-GAL4>mitoGFP; drp1^+^* (E, *drp1^+^*), *PGAM5^1^/Y; MHC-GAL4>mitoGFP; drp1^+^* (F, *drp1^+^*; *PGAM5^1^*).

Evolutionarily-conserved GTPases Mfn and OPA1 promote the mitochondrial fusion event while another GTPase Drp1 regulates the mitochondrial fission [27], [28]. To determine the role of PGAM5 in the mitochondrial fission pathway, we manipulated the activities of the genes that are involved in mitochondrial fission/fusion in *dPGAM5* null flies. Decreased Mfn activity resulted in fragmented mitochondria in the indirect muscle tissues, which was not affected by removal of the *dPGAM5* gene (Figure 4A–4D and 4G, Figure S2). Conversely, an increased mitochondrial fission activity by introducing an extra copy of the *drp1* gene was not suppressed in the *dPGAM5* null genetic background (Figure 4E–4G). These results suggested that dPGAM5 may function upstream of Mfn or Drp1, or that the mechanism of the mitochondrial morphological changes by dPGAM5 is independent of that of the known fusion/fission components.

### dPGAM5 Modulates Phenotypes Caused by *dPINK1* Inactivation in *Drosophila*

**Figure 5:**
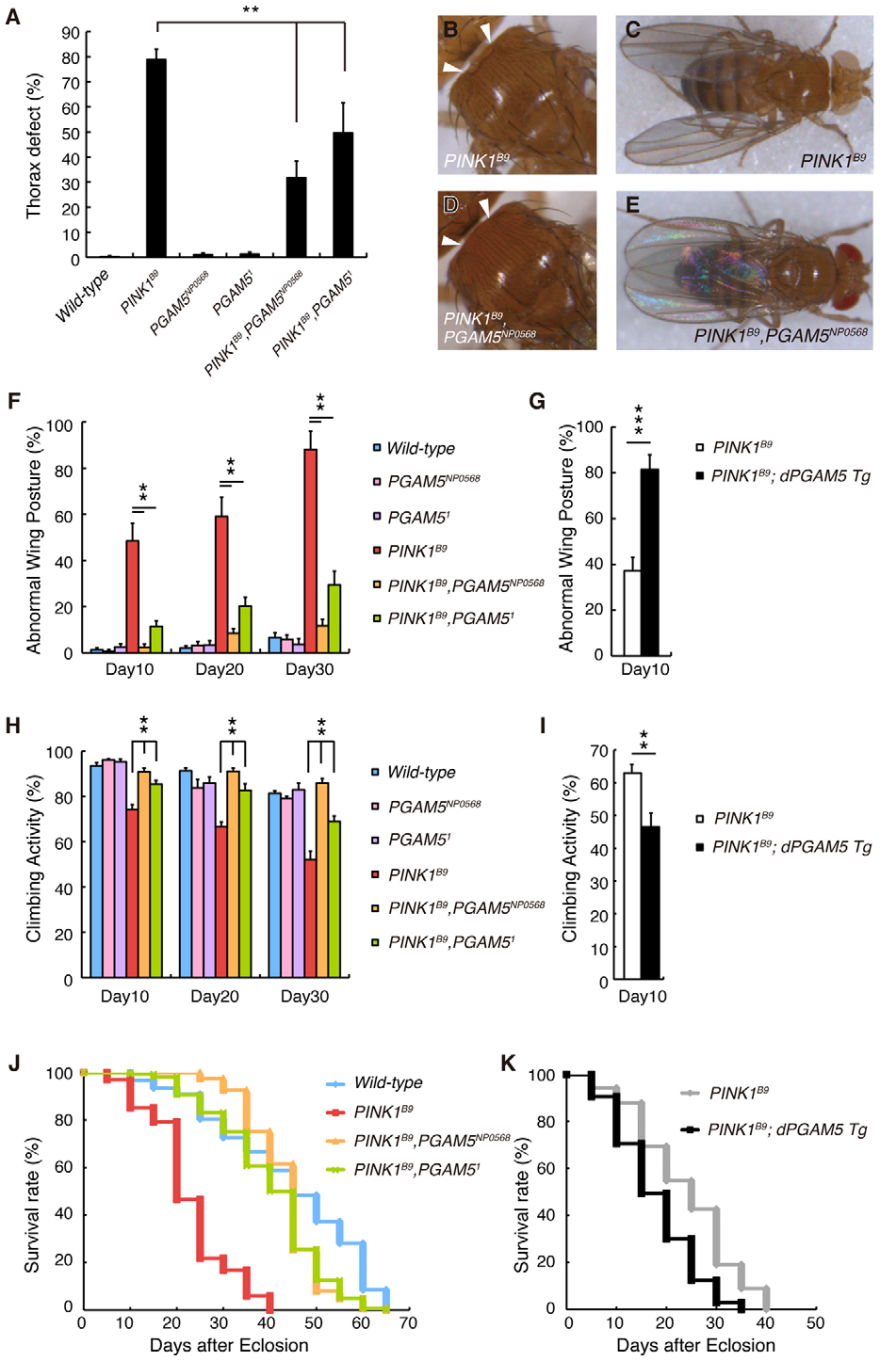
Loss of *dPGAM5* suppresses *dPINK1* mutant phenotypes in *Drosophila*. A thorax defect (B, arrowheads) and abnormal wing posture (C) caused by loss of *dPINK1* activity are suppressed in *dPGAM5* mutant genetic backgrounds (A, D and E). (F) Percentage of 10-, 20- and 30-day-old male flies showing abnormal wing postures. Error bars show S.E. from three experiments. (G) Percentage of 10-day-old male *PINK1^B9^* and *PINK1^B9^* ubiquitously overexpressing *dPGAM5* flies showing abnormal wing postures. Error bars show S.E. from three experiments. (H, I) Percentage of locomotor activity. Error bars show S.E. from three repeated experiments. (J) Lifespan of adult male flies. Loss of *dPGAM5* partially improved the reduced lifespan seen in *PINK1^B9^* fly (*PINK1^B9^ vs. PINK1^B9^*, *PGAM5^NP0568^* or *PINK1^B9^*, *PGAM5^1^*, *p*<0.001; *wild-type vs. PINK1^B9^*, *PGAM5^NP0568^* or *PINK1^B9^*, *PGAM5^1^*, *p*<0.01 by the log rank test). (K) Lifespan of adult male *PINK1^B9^* and *PINK1^B9^* ubiquitously overexpressing *dPGAM5* flies. Overexpression of dPGAM5 further reduced the lifespan (*PINK1^B9^ vs. PINK1^B9^*; *dPGAM5 Tg*, *p*<0.001). The same files were used in (A–F, H and J) and in (G, I and K). The genotypes and the number used in the assays are; *wild-type* (*PINK1^RV^*/*Y*, *n* = 161), *PGAM5^NP0568^* (*PGAM5^NP0568^*/*Y*, *n* = 161), *PGAM5^1^* (*PGAM5^1^*/*Y*, *n* = 161), *PINK1^B9^* (*PINK1^B9^*/*Y*, *n* = 101), *PINK1^B9^*, *PGAM5^NP0568^* (*PINK1^B9^*, *PGAM5^NP0568^*/*Y*, *n* = 162) and *PINK1^B9^*, *PGAM5^1^* (*PINK1^B9^*, *PGAM5^1^*/*Y*, *n* = 160) in (A–F, H and J), *PINK1^B9^* (*PINK1^B9^*/*Y; Da-GAL4/+*, *n* = 162) and *PINK1^B9^*, *dPGAM5 Tg* (*PINK1^B9^*/*Y; Da-GAL4> UAS-dPGAM5*, *n* = 161) in (G, I and K).

**Figure 6:**
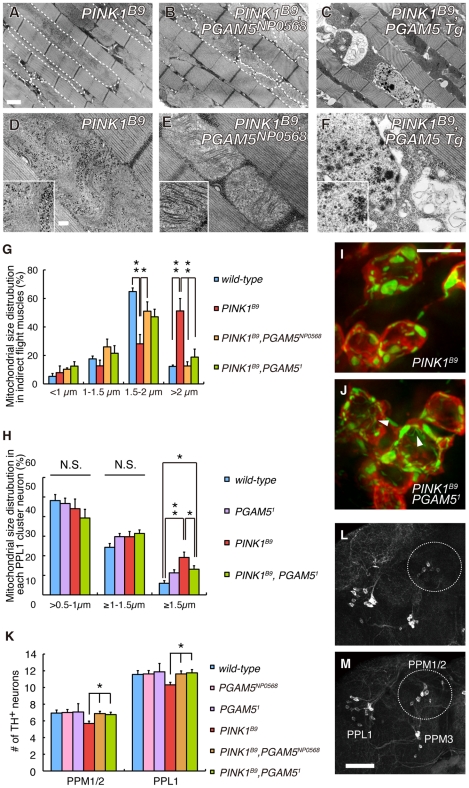
Loss of *dPGAM5* improves degeneration of the mitochondria and DA neurons caused by *dPINK1* inactivation in *Drosophila*. (A–F) TEM analysis of the indirect flight muscle and morphology of mitochondria in 2-day-old adult flies with the indicated genotypes. In A and B, some mitochondria are outlined with broken lines. The insets in D–F show representative mitochondria matrixes. Scale bars  = 1 µm in A–C and 200 nm in D–F. (G) Quantification of the percentage of mitochondrial size distribution in the indirect muscle tissue from *wild-type* (*n* = 136 from 5 adult flies), *PINK1^B9^* (*n* = 96 from 5), *PINK1^B9^*, *PGAM5^NP0568^* (*n* = 116 from 5), *PINK1^B9^*, *PGAM5^1^* (*n* = 111 from 5) as shown in Figure 3K. Data are shown as means ± SE (* *p*<0.05, **p<0.01). (H–J) Quantification of the percentage of cytoplasmic mitochondrial aggregates with diameter of 0.5–1.0, 1.0–1.5 or ≥1.5 µm in each PPL1 TH^+^ neuron from *wild-type* (*n* = 373 from 18 adult flies), *PGAM5^1^* (*n* = 356 from 18), *PINK1^B9^* (*n* = 231 from 11), *PINK1^B9^PGAM5^1^* flies (*n* = 235 from 13). Mitochondrial morphology was revealed by mitoGFP as shown in Figure 3L–3O. Data are shown as means ± SE (*, *p*<0.05;**, *p*<0.01; N.S., not significant). Tubular or reticular mitochondria were excluded from the estimation due to difficulty in the counting. However, the ratio of mitochondria with that morphology was also increased in *PINK1^B9^PGAM5^1^* flies (J) compared with that in *PINK1^B9^* flies (I). Arrowheads in (J) indicate representative tubular or reticular mitochondria. Scale bar in (I)  = 5 µm. (K) Quantification of TH^+^ DA neuron number in the PPM1, PPM2 and PPL1 clusters in 25-day-old males. PPM1 and PPM2 cluster neurons were counted together. Data are shown as means ± SE (*, *p*<0.05; *n* = 16). (L, M) Representative images of PPM1/2, PPM3 and PPL1 clusters of *PINK1^B9^* (L) and *PINK1^B9^*, *PGAM5^NP0568^* flies (M) visualized with anti-TH antibody. Scale bar in (M)  = 50 µm. The genotypes are: *PINK1^RV^/Y* (*wild-type*), *PINK1^B9^/Y* (*PINK1^B9^*), *PGAM5^NP0568^/Y* (*PGAM5^NP0568^*), *PGAM5^1^/Y* (*PGAM5^1^*), *PINK1^B9^*, *PGAM5^NP0568^/Y* (*PINK1^B9^*, *PGAM5^NP0568^*), *PINK1^B9^*, *PGAM5^1^/Y* (*PINK1^B9^*, *PGAM5^1^*), *PINK1^B9^/Y*; *Da-GAL4*> *dPGAM5* (*PINK1^B9^*, *PGAM5 Tg*), in (A–G, K–M), *PINK1^RV^/Y; TH-GAL4> mitoGFP* (*wild-type*), *PINK1^B9^/Y; TH-GAL4> mitoGFP* (*PINK1^B9^*), *PGAM5^1^/Y; TH-GAL4> mitoGFP* (*PGAM5^1^*), *PINK1^B9^*, *PGAM5^1^/Y; TH-GAL4> mitoGFP* (*PINK1^B9^*, *PGAM5^1^*) in (H–J).

We next confirmed that the results of the genetic tests in Figure 1B and 1C using a LOF allele for *dPINK1*, *PINK1^B9^* to exclude off-target effects due to RNAi (Figure 5). Adult *PINK1^B9^* flies often have abnormal thoraces with dents in the mid-anterior region, which is likely due to degeneration of the muscle tissues lining the inside of the thorax (Figure 5B) [8]. This thorax phenotype seen in *PINK1^B9^* flies can be suppressed by introduction of the *PGAM5^NP0568^* or the *PGAM5^1^* allele (Figure 5A and 5D). We then examined the effects of *dPGAM5* inactivation on *dPINK1* mutant phenotypes that progressively increase over time. As described above, loss of *dPINK1* activity leads to the appearance of abnormal wing postures, which is indicative of flight muscle degeneration, and the percent of affected flies increases with advancing age (Figure 5C and 5F) [8]. Introduction of the *dPGAM5* mutant alleles dramatically suppresses this phenotype (Figure 5E and 5F), whereas ectopic expression of dPGAM5 enhances the phenotype (Figure 5G). Progressive loss of climbing ability and the shorter lifespan of *PINK1^B9^* flies are additional prominent phenotypes that may represent dysfunction of DA neurons of the central nervous system and muscle degeneration. The *dPGAM5* mutant alleles also significantly improved these phenotypes (Figure 5H and 5J). Conversely, overexpression of dPGAM5 worsened the phenotypes (Figure 5I and 5K). Transmission electron microscopy (TEM) sections from one day-old adult *PINK1^B9^* mutant flies reveal that mitochondria in the indirect flight muscles are abnormally fused with one another and that the structures of the mitochondrial cristae are unclear (*i.e.* the cristae have lost the normal electron density seen by TEM) as compared to those of a *dPINK1* revertant line (Figure 6A and 6D compared to Figure 3C and 3G). Importantly, the mitochondrial hyperfusion and loss of cristae usually observed in *dPINK1* mutant animals can be partly suppressed by introduction of the *PGAM5^NP0568^* or the *PGAM5^1^* allele (Figure 6B, 6E, and 6G). In sharp contrast, transgenic expression of dPGAM5 further promoted mitochondrial degeneration (Figure 6C and 6F). Similar results were obtained when mitochondria in DA neurons of the adult brain (Figure 6H, 6I, and 6J) and in the indirect muscle tissues (Figure S3) were visualized using a version of GFP with a mitochondrial targeting signal (mitoGFP). Mitochondrial morphology in DA neurons in wild-type flies showed a long tubular network in the cytoplasm (Figure 3L). As previously reported, DA neurons in *PINK1^B9^* flies form spherical aggregates of mitochondria (Figure 6I). Removal of *dPGAM5* from *PINK1^B9^* flies led to an increase in the number of small fragmented or tubular mitochondria (Figure 6J). These results suggest that excessive mitochondrial aggregation, which is modulated by dPGAM5 inactivation, is indicative of a functional failure of mitochondria in DA neurons.

Consistent with the beneficial effects of dPGAM5 inactivation on the mitochondrial degeneration seen in *PINK1^B9^* flies, we observed that dPGAM5 inactivation suppresses the loss of DA neurons in the protocerebral posterior lateral 1 (PPL1) and protocerebral posterior medial 1 and 2 (PPM1/2) clusters of aged flies (Figure 6K–6M).

### Removal of *dPGAM5* Fails to Suppress Phenotypes Resulting from *dparkin* Inactivation

**Figure 7:**
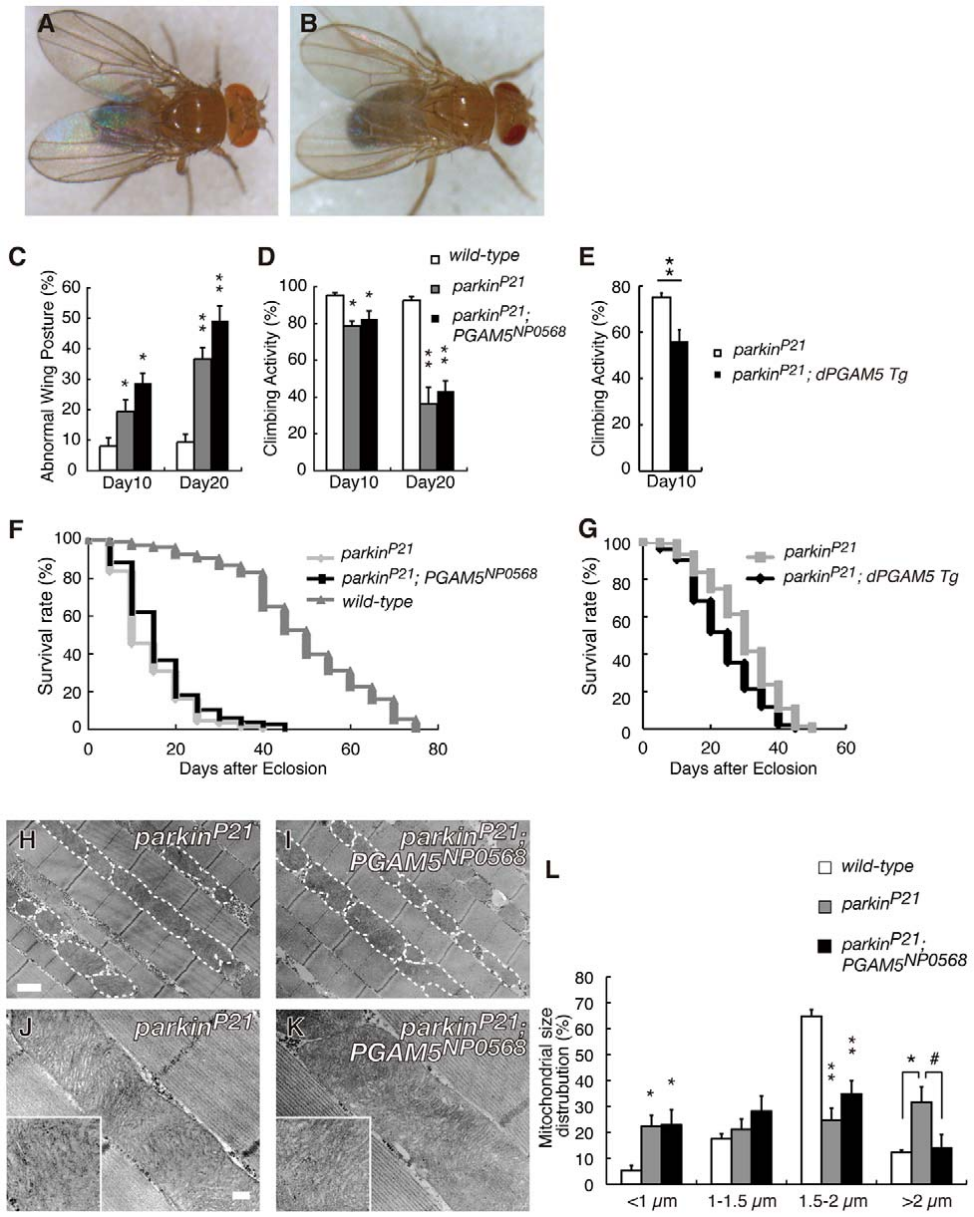
Disruption of *dPGAM5* fails to suppress the mitochondrial phenotype caused by *dParkin* inactivation in *Drosophila*. The abnormal wing posture caused by a homozygous *dParkin* mutation (A) was not suppressed by removal of the *dPGAM5* gene (B). (C) Percentage of flies with abnormal wing posture among 10- and 20-day-old male *wild-type* (*n* = 105), *parkin^P21^* (*n* = 102) and *PGAM5^NP0568^; parkin^P21^* (*n* = 109) flies. Error bars show S.E. from three repeated experiments. *, *p*<0.05; **, *p*<0.01 *vs. dParkin*(+/+). (D) Percentage of flies showing locomotor activity among 10- and 20-day-old male *parkin^P21^* (*n* = 86), *parkin^P21^* (*n* = 73) and *PGAM5^NP0568^; parkin^P21^* (*n* = 78) flies. Error bars show S.E. from twenty repeated experiments. *, *p*<0.05; **, *p*<0.01 *vs. dParkin*(+/+). (E) Locomotor activity of 10-day-old male *parkin^P21^* (*n* = 153) and *parkin^P21^* ubiquitously overexpressing *dPGAM5* flies (*parkin^P21^; dPGAM5 Tg*, *n* = 155) flies. Error bars show S.E. from twenty repeated experiments. **, *p*<0.01. (F) Lifespan of adult male *wild-type* (*n* = 104), *parkin^P21^* (*n* = 102) and *PGAM5^NP0568^; parkin^P21^* (*n* = 91) flies. *PGAM5^NP0568^; parkin^P21^ vs. parkin^P21^*, *p* = 0.191; *wild-type vs. parkin^P21^*, *p*<0.001 by log rank test. (G) Lifespan of adult male *parkin^P21^* (*n* = 153) and *parkin^P21^; dPGAM5 Tg* flies (*n* = 155) flies. *parkin^P21^ vs. parkin^P21^; dPGAM5 Tg*, *p*<0.001 by log rank test. (H–K) TEM analysis of the indirect flight muscle and mitochondrial morphology in tissue from flies of the indicated genotypes. The long tubular mitochondrial phenotype seen in *parkin^P21^* flies can be rescued by *dPGAM5* inactivation (H and I). However, the mitochondrial matrix still appears degenerated (insets in J and K). Scale bars  = 1 µm in H and I and 200 nm in J and K. (L) Quantification of the percentage of mitochondrial size distribution in the indirect muscle tissue from *wild-type* (*n* = 136 from 5 adult flies), *parkin^P21^* (*n* = 89 from 5) and *parkin^P21^; PGAM5^NP0568^* flies (*n* = 84 from 5) as shown in (H–K). The length of the mitochondria in the direction of the myofibrils was measured. Data are shown as means ± SE (* *p*<0.05, **p<0.01 *vs. wild-type*; # *p*<0.05 *vs. parkin^P21^; PGAM5^NP0568^*). The genotypes are: *+/Y* (*wild-type*), *+/Y; parkin^P21^/parkin^P21^* (*parkin^P21^*), *PGAM5^NP0568^/Y; parkin^P21^/parkin^P21^* (*parkin^P21^; PGAM5^NP0568^*).

Previous studies in *Drosophila* suggested that *dPINK1* is genetically associated with *dparkin* and furthermore, that *dPINK1* functions upstream of *dparkin* [7]–[9]. In addition, *dparkin* null mutations cause mitochondrial degeneration of a subset of tissues in *Drosophila*, which phenocopies dPINK1 inactivation [29], [30]. Given the evidence that PGAM5 is involved in the PINK1 pathway, we next asked if dPGAM5 also affects the *in vivo* mitochondrial phenotypes associated with mutations in *dParkin*. Introduction of *PGAM5^NP0568^* in the *parkin* hypomorphic genetic background (*parkin^P21^*) had little effect on abnormal wing postures (Figure 7A–7C) [29], [30]. Consistent with the result in the wing phenotype, loss of *dPGAM5* activity failed to rescue the age-dependent motor defects and shorter lifespan observed in *parkin^P21^*flies (Figure 7D and 7F). In the same settings, overexpression of dPGAM5 further enhanced both motor defect and reduced lifespan phenotype (Figure 7E and 7G). Loss of dParkin activity results in an elongated morphology in mitochondria of the adult indirect flight muscle tissues, a phenotype that was suppressed by loss of the *dPGAM5* gene (Figure 7H, 7I, and 7L). However, the crista structures of the mitochondria were not restored by inactivation of the *dPGAM5* gene (Figure 7J and 7K). Taken together, these data suggest that *dPGAM5* lies genetically upstream of *dparkin*, or functions independently of *dparkin* downstream of *dPINK1* in *Drosophila*.

### Activation of a Redox Control Pathway Improves Viability of *dPINK1* Mutant Flies

**Figure 8:**
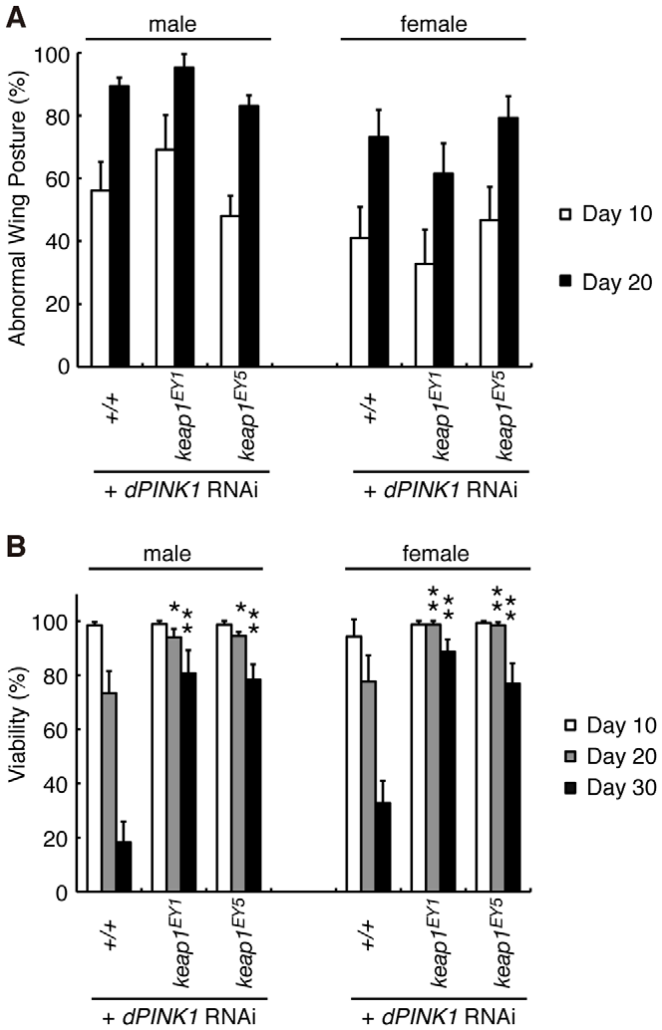
Reduction of Keap1 activity improves the lifespan of *dPINK1* RNAi flies. Removal of one copy of *Drosophila keap1* had no effects on the wing phenotype of *dPINK1* RNAi flies (A) but improved viability (B). *, *p*<0.05; **, *p*<0.01 *vs.* age-matched *dPINK1* RNAi group. The genotypes are as follows: *MHC-GAL4> dPINK1^RNAi^* (+/+), *MHC-GAL4> dPINK1^RNAi^/Keap^EY1^* (*Keap^EY1^*), *MHC-GAL4> dPINK1^RNAi^/Keap^EY5^* (*Keap^EY5^*). Flies were raised at 29°C.

PGAM5 was previously reported to interact with Keap1 (Gene ID: 9817), a substrate adaptor protein for a Cullin-3-dependent E3 complex [17]. In a normal redox state, the Keap1 complex suppresses activity of a bZIP transcription factor, Nrf2, through ubiquitin/proteasome-dependent protein degradation [31]. Oxidative stress impairs inhibition of Nrf2 by Keap1 [31]. Nrf2 thus becomes stabilized and activates oxidative stress protective genes, restoring cellular redox homeostasis. Although we confirmed the association of PGAM5 with Keap1 in human cultured cells, the proposed Keap1-binding motif in PGAM5, N*X*ESGE, was not conserved in dPGAM5 (Figure S1). On the other hand, Keap1/Nrf2 signaling does appear to be conserved in *Drosophila* [32]. We tested if Keap1/Nrf2 signaling modulates *PINK1* phenotypes. Removal of a copy of the *keap1* gene (Gene ID: 42062) in *dPINK1* knockdown flies, wherein the *dPINK1* RNAi was expressed in the muscle tissues, failed to rescue the abnormal wing posture (Figure 8A). However, *Keap1* heterozygosity is beneficial to survival of aging *dPINK1* knockdown files, supporting a previous report suggesting that oxidative stress is partly involved in the PINK1 pathology (Figure 8B) [33], [34].

## Discussion

The event of fusion/fission is required for maintenance of a healthy mitochondrial population. Mitochondrial fusion is believed to require the interchange of a set of internal components, including copies of the mitochondrial genome, respiratory proteins and metabolic products. Mitochondrial fission has been proposed to play a role in disposal of damaged mitochondria, such as those with a reduced mitochondrial membrane potential, via mitophagy [35]. A role for PINK1 in the regulation of mitochondrial fission/fusion dynamics has recently been demonstrated in *Drosophila* [10]–[12]. The PINK1/Parkin pathway appears to promote fission and/or inhibits fusion, likely through an indirect mechanism. Indeed, loss of dPINK1 or dParkin produces swollen or enlarged mitochondria in tissues with high-energy demands, such as the muscles, which is suppressed by reduced fusion activity or increased fission activity after genetic manipulation of the mitochondrial fission/fusion machinery. Namely, either reducing the activity of the mitochondrial fusion proteins OPA1 and Mfn, or increasing the activity of a mitochondrial fission protein, Drp1, can partially rescue *PINK1* and *parkin* mutant phenotypes.

We identified PGAM5 as a PINK1-binding protein and went on to show that *dPGAM5* can modulate *dPINK1* mutant phenotypes. Loss of *dPGAM5* activity had little effect on the lifespan of a *dPINK1* RNAi fly strain in our initial *in vivo* test (Figure 1C). However, we found that loss of *dPGAM5* does significantly extend lifespan of *PINK1^B9^* mutant flies (Figure 5J). We speculate that continuous expression of the short hairpin RNA in the RNAi-based test confers additional toxicity, leading to a shorter lifespan in a sequence-independent manner, such that the suppressive effect of *dPGAM5* mutations cannot be detected in the *PINK1* RNAi flies.

dPGAM5 appears to be dispensable for mitochondrial homeostasis in *Drosophila*, as overall, flies homozygous for a null allele of *dPGAM5*, *PGAM5^1^*, appear to be normal. It has previously been reported that ectopic expression of PGAM5 leads to perinuclear aggregation or small fragmentation of mitochondria in mammalian cultured cells, which suggested that PGAM5 has a role in regulation of mitochondrial fission/fusion process or mobility [24]. Our study also observed alteration of mitochondrial morphology in *Drosophila* with different dPGAM5 activities. Transgenic expression of dPGAM5 or dPGAM5-2 leads to fragmentation of mitochondria both in the TH^+^ neurons and indirect flight muscles (Figure 3E, 3F, 3I and 3J). By contrast, dPGAM5 LOF moderately increases mitochondria with a longer tubular or a swollen morphology (Figure 3D and 3K). Our genetic tests with the known mitochondrial fusion/fission machinery suggested that PGAM5 acts upstream of them or in an independent pathway (Figure 4). Given that PGAM5 is involved in mitochondrial fission, loss of PGAM5 would be expected to enhance the *PINK1* mutant phenotype in *Drosophila*, similar to the interaction between *PINK1* and the mitochondrial fusion/fission machinery [10]–[12]. Interestingly, the number of large aggregated mitochondria, which are frequently seen in *PINK1^B9^* flies, was mildly decreased in TH^+^ neurons of *PINK1^B9^PGAM5^1^* flies (Figure 6H–6J). Moreover, loss of dPGAM5 also modulated the mitochondrial morphology of *dParkin* mutant fly without suppressing the mitochondrial degeneration (Figure 7H–7L). Based on these observations, it could be speculated that PGAM5 does not directly regulate mitochondrial fission but instead, modulates the PINK1 pathway in a different way. Recent studies have proposed two different models for the mechanism of mitochondrial morphological changes through the PINK1/Parkin pathway in *Drosophila* and mammals. Ziviani *et al.* and Poole *et al.* have demonstrated that dParkin promotes degradation of Mfn in a dPINK1-dependent manner, which leads to mitochondrial fragmentation in *Drosophila* [16], [36]. Our current results demonstrated that the loss of dPGAM5 activity does not affect mitochondrial fragmentation caused by reduction of Mfn activity, suggesting that dPGAM5 might not contribute to the proposed PINK1/Parkin pathway (Figure 4). Sandebring *et al.* have proposed that accumulation of damaged mitochondria by PINK1 inactivation results in mitochondrial calcium efflux, which activates Drp1 through Calcineurin-mediated dephosphorylation of Drp1 in human cells [37]. This model well explains the observation that loss of PINK1 indirectly promotes mitochondrial fragmentation in mammalian cells and the indication that PINK1 is not a core component of the fusion/fission machinery in a *Drosophila* study [11]. However, most of *Drosophila* studies do not support a result that loss of PINK1 leads to mitochondrial fragmentation in mammals. Thus, it still remains a question for further investigation how PGAM5 modulates the mitochondrial dynamics.

**Figure 9:**
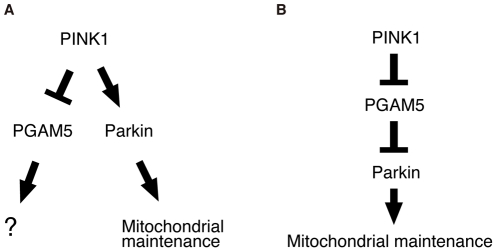
Schematic of the proposed PINK1/PGAM5 pathways in *Drosophila*. (A) PGAM5 has a role in mitochondrial activities independently of Parkin downstream of PINK1. (B) PGAM5 negatively regulates Parkin downstream of PINK1.

Although the property of PGAM5 to physically interact with PINK1 appears to be conserved between human and *Drosophila*, the functional significance of this binding remains to be established. PINK1 and PGAM5 have kinase and phosphatase activities, respectively. However, there is no evidence to suggest that PINK1 directly phosphorylates PGAM5, or that PGAM5 dephosphorylates PINK1 (Figure 2G and 2H) [18], [25], [26]. The PINK1 protein levels are maintained at very low level under steady-state conditions by constitutive processing and subsequent degradation through the ubiquitin-proteasome pathway [15]. Recent studies suggested that PINK1 selectively translocates from cytosol to mitochondria with low membrane potential, at which PINK1 is stabilized [14], [15], [38]. The accumulated PINK1 on the depolarized mitochondria further recruits Parkin to induce mitophagy [13]–[15], [38]. However, Parkin does not seem to be the target of PINK1 kinase activity [15], and PINK1 does not seem to activate Parkin E3 activity directly (data not shown). Based on these findings, it is possible that PGAM5 may promote a selective recruitment of PINK1 to the outer membrane of the damaged mitochondria, or that PGAM5 may regulate PINK1 stabilization. Our molecular analysis, however, did not support the idea that PGAM5 stabilizes PINK1 (data not shown). In addition, because loss of *dPGAM5* partially suppresses *dPINK1* null phenotypes, it seems likely that PINK1 negatively regulates PGAM5 function (Figure 9). PGAM5 was originally identified as a Bcl-xL-binding protein, and in itself can be toxic to cells, promoting mitochondrial fragmentation, when expressed at high levels (Figure 3B). Therefore, it seems possible that PGAM5 modulates a cell protective or a mitochondrial morphogenetic activity of the Bcl-2 family member Bcl-xL downstream of PINK1 but in a pathway that is independent from Parkin (Figure 9A) [39]. In this context, PINK1 may suppress the cell toxic action of PGAM5 through an indirect mechanism where an unidentified substrate of PINK1 inactivates PGAM5. Interestingly, a recent report suggests that the *dPINK1* phenotype can be partially suppressed by transgenic expression of *Drosophila* Bcl-2 protein Buffy [8]. Alternatively, PGAM5 may be one of components of a negative regulator complex against Parkin E3, downstream of PINK1 (Figure 9B). Matsuda *et al.* have reported that Parkin E3 activity is activated only at the depolarized mitochondria, suggesting the existence of its negative regulator(s) [38]. E3 activity of Parkin may be released when PINK1 associates with the negative regulator complex via PGAM5 and suppresses its function by phosphorylation of another complex component(s). This idea might be partly supported by our observation that loss of *dPGAM5* had little effect on *dparkin* mutant flies.

Although the primary cause of the mitochondrial degeneration by the loss of *PINK1* remains less obvious, the growing evidence suggests that PINK1 eliminates oxidatively damaged mitochondria in cooperation with Parkin, the failure of which leads to tissue degeneration. Supporting for this hypothesis, the mitochondrial phenotypes of *dPINK1* and *dParkin* mutant flies are primarily exhibited in similar tissues that require higher energy demands [7]–[9]. Although dPAGM5 might not regulate Keap1 function in *Drosophila*, the Keap1/Nrf2 pathway appears to be conserved in *Drosophila* [32], and activation of the Keap1/Nrf2 pathway by genetic manipulation effectively suppressed the short-lifespan phenotype by *dPINK1* inactivation. This finding may also support the above idea that the accumulation of oxidatively damaged mitochondria leads to degeneration of specific tissues, providing a hint of therapeutic strategies for *PINK1*-associated PD.

In conclusion, the results of our genetic study demonstrate that the mitochondrial-localized protein PGAM5 modulates the PINK1 pathway in *Drosophila*. However, further work will be required to determine how PGAM5 regulates the PINK1 pathway at the molecular level, as well as to determine if manipulation of PGAM5 activity might provide a therapeutic advantage in treatment of *PINK1*-associated PD.

## Materials and Methods

### Purification of PINK1-Binding Proteins

HEK293 cells stably expressing hPINK1-FLAG or parent cells were grown in suspension culture (Joklik-modified Eagle's minimum essential medium with 5% fetal bovine serum). The cell pellet (2.4×10^8^ cells) was homogenized in lysis buffer (50 mM Tris pH 7.4, 120 mM NaCl, 5 mM EDTA, 10% glycerol, 1% Trion-X100) supplemented with Complete Protease Inhibitors (Roche Diagnostics). The soluble fraction of the suspension was immunoprecipitated with anti-FLAG M2 agarose (Sigma-Aldrich) and washed five times in lysis buffer. The fractions eluted with 200 µg/ml 3x FLAG peptide were resolved by SDS-PAGE. Specific bands detected by silver staining were excised for in-gel digestion. The digest extracted from the gel was subjected to online HPLC-MS/MS, followed by informatics-based identification of the proteins (Nippon Proteomics).

### *Drosophila* Genetics

Fly culture and crosses were performed on standard fly food containing yeast, cornmeal and molasses, and flies were raised at 25°C unless otherwise indicated. To generate *UAS-dPGAM5* transgenic lines, cDNA for *dPGAM5* and *dPGAM5-2* obtained by RT-PCR from adult *Drosophila* total RNA was subcloned into the *pUAST* vector. Introduction of transgenes into *Drosophila* germ line and establishment of transgenic lines into a *w^–^* background were performed by BestGene Inc. (Chino Hills, CA). A *P*-element insertion line for *dPGAM5* mutant, *PGAM5^NP0568^* obtained from Kyoto *Drosophila* Genetic Resource Center, expresses a reduced level (∼25%) of mRNA, in which *NP0568* element is integrated 50 bp downstream of the *dPGAM5* translational start site (Figure S1B). We could not detect any dPGAM5 protein signal in *PGAM5^NP0568^* homozygous flies (Figure S1C). The *PGAM5^NP0568^* line was backcrossed to *w^–^* for six generations to remove background mutations, and used for most experiments as a *dPGAM5* mutant line. *UAS-dPGAM5* RNAi (VDRC 51655) and *UAS-mfn* RNAi (VDRC105261 and VDRC40478) strains were obtained from the Vienna *Drosophila* RNAi Center. To generate *PGAM5^1^*, the KG09727 *P*-element insertion (obtained from the Bloomington *Drosophila* Stock Center) was mobilized using Δ*2*–*3* transposase, and the entire dPGAM5 coding region was deleted by imprecise excision, as shown in Figure S1A. All other fly stocks and *GAL4* lines used in this study were obtained from the Bloomington *Drosophila* Stock Center and have been previously described: *UAS-dPINK1 RNAi* [9]; *PINK1^B9^* and revertant *PINK1^RV^* [8]; *parkin^P21^* [30]; *keap1^EY1^*and *keap1^EY5^* [32]; *Drp1^2^* and *Drp1^+^* [40].

### RT-PCR and Plasmids

For quantitative RT-PCR analysis, reverse transcription and PCR reactions with total RNA extracted from fly heads were performed using a Superscript VILO cDNA synthesis kit (Invitrogen) and SYBR GreenER qPCR SuperMix (Invitrogen), respectively. Full-length cDNAs corresponding to *hPGAM5* (GenBank NP_001170543) and a short isoform of *hPGAM5* (hPGAM5-S, GenBank NM_138575) were amplified by RT-PCR from total RNA purified from HEK293 cells or a cDNA clone (RIKEN clone ID: IRAK003D15), and was cloned in *pcDNA3-Myc*, *pGEX6P-1* and *pGEX4T-1* vectors. Expression plasmids for *hPINK1-FLAG* and *pUAST-dPINK1-Myc* have been reported elsewhere [9], [20].

### Antibodies

Rabbit anti-human PGAM5 polyclonal antibody was raised against recombinant GST-tagged PGAM5 domain (89–289 aa) produced in the *E. coli* strain BL21(DE3)pLysS (Novagen), and was affinity-purified against the antigen. Rabbit anti-dPGAM5 polyclonal antibody was raised against the peptide ELLTNRIPRDVKNVV. Anti-hPINK1 antibody (BC100-494), anti-Myc (4A6) and anti-FLAG (M2) antibodies were purchased from Novus, Millipore and Sigma-Aldrich, respectively. Mouse anti-TH monoclonal antibody was purchased from ImmunoStar, and rabbit anti-*Drosophila* TH polyclonal antibody was described previously [9].

### Cell Culture, Immunoprecipitation, and Immunoblot Analysis

Transfection of mammalian cultured cell, immunopurification of FLAG-protein from transfected cell lysate and immunoblot analysis was performed as described previously [4], [41]. For the hPGAM5 RNAi experiment, HEK293 cell lysate transfected with 20 µM stealth RNAi reagent against hPGAM5 or a control RNAi reagent (Invitrogen), was analyzed 72 hrs after transfection. To detect an endogenous interaction between PINK1 and PGAM5, we treated HEK293 cells (2×10^7^ cells) with 20 µM carbonyl cyanide 3-chlorophenylhydrazone for 24 hrs to induce a sufficient level of human PINK1 protein for the study. The treated cells were subjected to immunoprecipitation using a Rabbit TrueBlot kit combined with rabbit anti-human PINK1 or rabbit anti-Delta (Santa Cruz) as a species-matched control. For the preparation of fly samples for immunoblot analysis, fly heads were directly homogenized in 20 µl/head of SDS sample buffer using a motor-driven pestle. After centrifugation at 16,000 g for 10 min, the supernatant was used in SDS-PAGE.

### *In Vitro* Phosphorylation Assay

Recombinant 2x GST-dPINK1 (153–709 aa), which has an N-terminal GST-tag and a C-terminal GST/6x His tag, was produced in the *E. coli* strain pG-KJE8/BL21 (TAKARA) and purified by a sequential purification with Ni-NTA agarose and glutathione sepharose. GST-hPGAM5-S (1–255 aa) and GST-hPGAM5 (1–289 aa) were incubated with 2x GST-dPINK1 as described in Figure 2.

### Whole-Mount Immunostaining and Transmission Electron Microscopic (TEM) Analysi*s*

Counting of TH-positive neurons was performed by whole-mount immunostaining of brain samples as described previously [9]. TEM images were obtained at the Biomedical Research Core of Tohoku University Graduate School of Medicine. All histochemical analyses were performed using DeltaVision microscope system (Applied Precision) or LSM5 PASCAL laser scanning microscope system (Carl Zeiss). The images obtained by DeltaVision system were deconvolved through 10 iterations using the DeltaVision deconvolution software (Applied Precision). Area calculation of the mitochondria was performed following established criteria for classification [12] using softWoRx (Applied Precision) or Image J software from the US National Institute of Health (http://rsb.info.nih.gov/ij/).

### Lifespan Assay and Quantification of Wing Phenotypes and Climbing Ability

For lifespan studies, twenty female adult flies per vial were maintained at 25°C, transferred to fresh fly food, and scored for survival every 4 or 5 days. To control for isogeny, the *PGAM5^NP0568^*, *PINK1^B9^* and *PGAM5^1^*, *PINK1^B9^* alleles were backcrossed to *PINK1^B9^* for six generations, and *parkin^P21^* and *PGAM5^NP0568^*; *parkin^P21^* were backcrossed to *w^−^* wild-type background for six generations, *UAS-dPGAM5* transgenic flies were generated in the *w^−^* genetic background and thus have matched genetic backgrounds. The lifespan of *PGAM5^1^* was compared in the *y^−^* genetic background. The number of flies exhibiting defective abnormal wing posture (held-up or drooped) was determined for each genotype [9]. A climbing assay was performed as described previously [42].

### Statistical Analysis

One-way repeated measures ANOVA was performed to determine significant differences among multiple groups unless otherwise indicated. If a significant result was achieved (*p*<0.05), the mean of the control and the specific test groups was analyzed using the Tukey-Kramer test. For lifespan assays, the Kaplan-Meier analysis with log-rank test was performed.

## Supporting Information

Figure S1 *dPGAM5* mutant alleles. (A) *PGAM5^NP0568^* and *PGAM5^1^* mutant alleles are depicted. Boxes, exons of the *dPGAM5* gene; triangles, the positions of the transposon *NP0568* and *KG09727* insertions. Coding regions and the transcript are depicted by black and yellow boxes, respectively. (B) Quantitative RT-PCR of the *dPGAM5* transcript in homozygous *dPGAM5* mutant and RNAi lines. Expression of the *dPINK1* RNAi was induced via the *Da*-*GAL4* driver. Primer-binding sites for PCR are shown as arrows in (A). (C) Immunoblot analysis of dPGAM5 in the homozygous *dPGAM5* mutant, RNAi and transgenic lines. LE, longer exposure. (D) Alignment of the amino acid sequences of PGAM5 orthologues. Putative transmembrane domains are underlined in green for mammalian PGAM5 and blue for *Drosophila* PGAM5. A red arrowhead indicates the point of insertion of the transposon *NP0568*. Red underlining, sequences corresponding to the reported keap1-binding motif in human PGAM5.
(0.69 MB TIF)

Figure S2 Quantitative RT-PCR of the *mfn* transcript in the *mfn* RNAi lines. Expression of the *mfn* RNAi was induced via the *Da*-*GAL4* driver, and total RNA was purified from 3^rd^ instar larvae because *mfn* RNAi flies exhibited a pupation-defect phenotype.
(0.18 MB TIF)

Figure S3 Loss of *dPGAM5* improved mitochondrial degeneration of the indirect flight muscles caused by dPINK1 inactivation. To visualize the mitochondria under a fluorescence microscopy, we used the *MHC-GAL4* driver to induce expression of a mitoGFP (green) transgene in 5-day-old adult flies with the indicated genotypes. Muscle tissue was counterstained with phalloidin (red). Integrity of the mitochondria in *PINK1^B9^* flies was partially restored by removal of *dPGAM5* as shown by recovery of the mitoGFP signal (green) in *PINK1^B9^PGAM5^1^* flies. The genotypes are as follows: *MHC-GAL4>MitoGFP* [*wild-type*], *PGAM5^1^/Y; MHC-GAL4>UAS-mitoGFP* [*PGAM5^1^*], *PINK1^B9^/Y; MHC-GAL4>UAS-mitoGFP* [*PINK1^B9^*], *PINK1^B9^*, *PGAM5^1^/Y; MHC-GAL4>UAS-mitoGFP* [*PINK1^B9^*, *PGAM5^1^*].
(1.40 MB TIF)
