# Supplementary figures and images for: The Loss of PGAM5 Suppresses the Mitochondrial Degeneration Caused by Inactivation of PINK1 in Drosophila
Source: PLoS Genet. 2010 Dec 2;6(12):e1001229. doi: 10.1371/journal.pgen.1001229 (PMC2996328; doi:10.1371/journal.pgen.1001229)

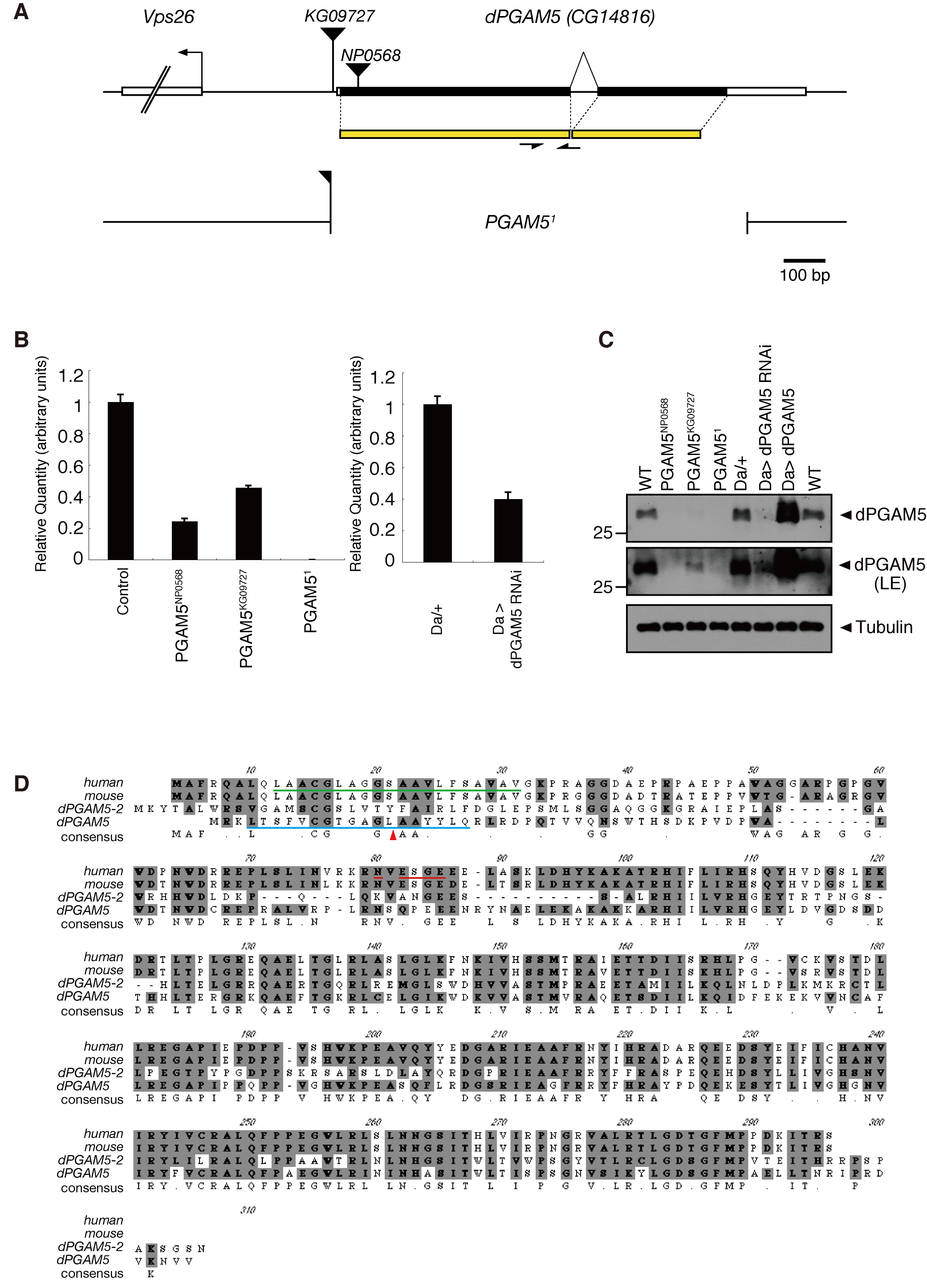

Supplement: Figure S1 — dPGAM5 mutant alleles. (A) PGAM5NP0568 and PGAM51 mutant alleles are depicted. Boxes, exons of the dPGAM5 gene; triangles, the positions of the transposon NP0568 and KG09727 insertions. Coding regions and the transcript are depicted by black and yellow boxes, respectively. (B) Quantitative RT-PCR of the dPGAM5 transcript in homozygous dPGAM5 mutant and RNAi lines. Expression of the dPINK1 RNAi was induced via the Da-GAL4 driver. Primer-binding sites for PCR are shown as arrows in (A). (C) Immunoblot analysis of dPGAM5 in the homozygous dPGAM5 mutant, RNAi and transgenic lines. LE, longer exposure. (D) Alignment of the amino acid sequences of PGAM5 orthologues. Putative transmembrane domains are underlined in green for mammalian PGAM5 and blue for Drosophila PGAM5. A red arrowhead indicates the point of insertion of the transposon NP0568. Red underlining, sequences corresponding to the reported keap1-binding motif in human PGAM5. (0.69 MB TIF) [file pgen.1001229.s001.tif]

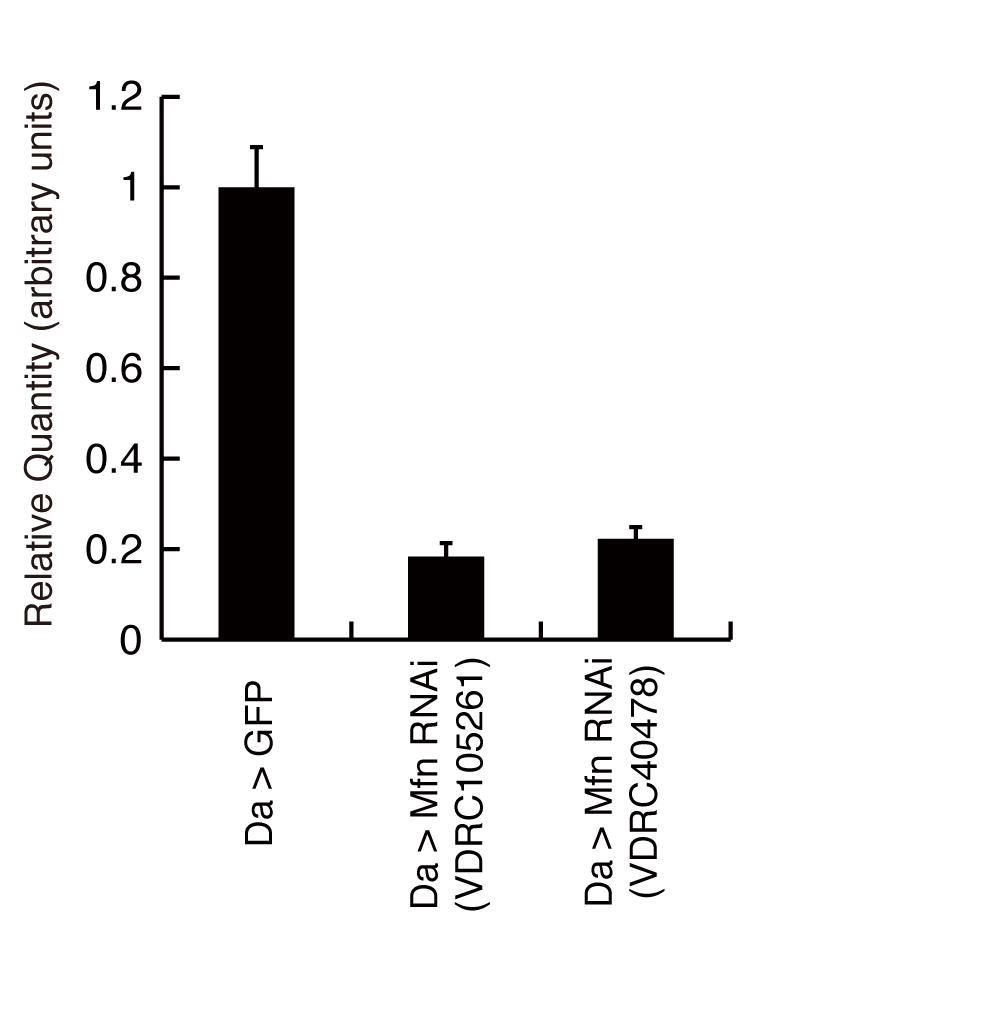

Supplement: Figure S2 — Quantitative RT-PCR of the mfn transcript in the mfn RNAi lines. Expression of the mfn RNAi was induced via the Da-GAL4 driver, and total RNA was purified from 3rd instar larvae because mfn RNAi flies exhibited a pupation-defect phenotype. (0.18 MB TIF) [file pgen.1001229.s002.tif]

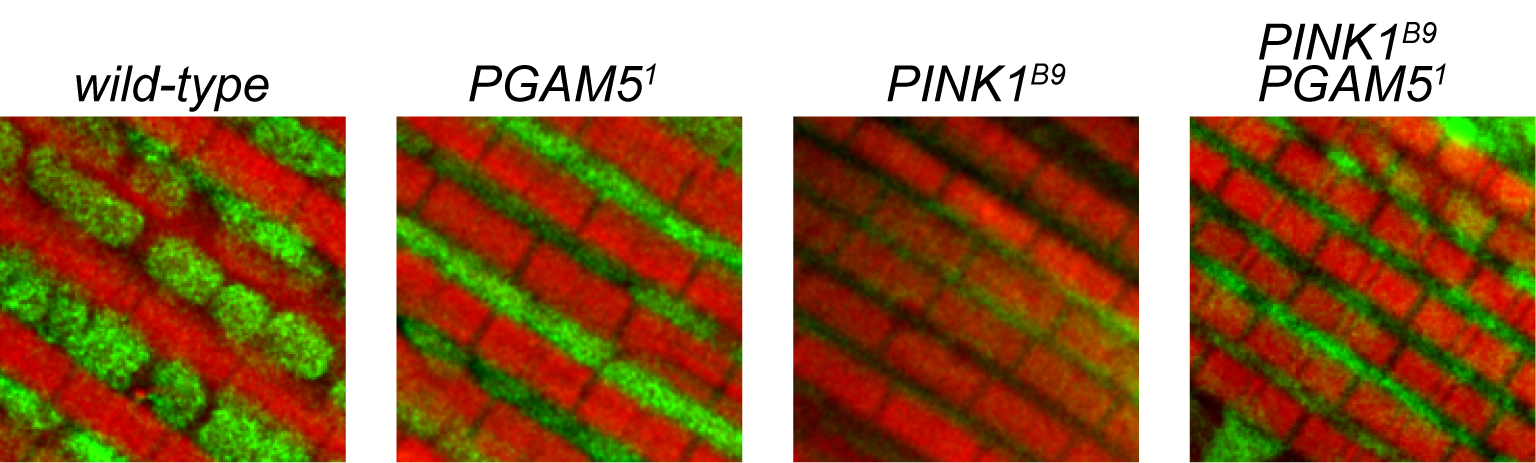

Supplement: Figure S3 — Loss of dPGAM5 improved mitochondrial degeneration of the indirect flight muscles caused by dPINK1 inactivation. To visualize the mitochondria under a fluorescence microscopy, we used the MHC-GAL4 driver to induce expression of a mitoGFP (green) transgene in 5-day-old adult flies with the indicated genotypes. Muscle tissue was counterstained with phalloidin (red). Integrity of the mitochondria in PINK1B9 flies was partially restored by removal of dPGAM5 as shown by recovery of the mitoGFP signal (green) in PINK1B9PGAM51 flies. The genotypes are as follows: MHC-GAL4>MitoGFP [wild-type], PGAM51/Y; MHC-GAL4>UAS-mitoGFP [PGAM51], PINK1B9/Y; MHC-GAL4>UAS-mitoGFP [PINK1B9], PINK1B9, PGAM51/Y; MHC-GAL4>UAS-mitoGFP [PINK1B9, PGAM51]. (1.40 MB TIF) [file pgen.1001229.s003.tif]
